# The Psychosocial Role of Body Image in the Quality of Life of Head and Neck Cancer Patients. What Does the Future Hold?—A Review of the Literature

**DOI:** 10.3390/medicina57101078

**Published:** 2021-10-09

**Authors:** Vlad Ioan Covrig, Diana Elena Lazăr, Victor Vlad Costan, Roxana Postolică, Beatrice Gabriela Ioan

**Affiliations:** 1 Doctoral School, Grigore T. Popa University of Medicine and Pharmacy, 700115 Iasi, Romania; vlad-ioan.d.covrig@d.umfiasi.ro; 2 Department of Oncology, Regional Institute of Oncology, 700483 Iasi, Romania; 3 Surgery Department, Oral and Maxillo-Facial Surgery, Faculty of Dentistry, Grigore T. Popa University of Medicine and Pharmacy, 700115 Iasi, Romania; victor.costan@umfiasi.ro; 4 Department of Psychology, Regional Institute of Oncology, 700483 Iasi, Romania; roxana.postolica@yahoo.com; 5 IIIrd Medical Department, Legal Medicine, Faculty of Medicine, Grigore T. Popa University of Medicine and Pharmacy, 700115 Iasi, Romania; beatrice.ioan@umfiasi.ro

**Keywords:** head and neck cancer, quality of life, body image, psychosocial, interventions

## Abstract

*Background and Objectives:* It is well known that among all cancers, cancers of the head and neck (HNC) have a major impact on patients’ quality of life. Disfigurement, anxiety and disabling physical and psychological symptoms affect people with HNC to such an extent that the suicide rate in this category of patients is exceeded only by that of patients with pancreatic cancer. The aim of this review was to summarize the published literature describing the severity of body image and quality of life impairment in patients with HNC over time, and to examine the psychosocial and functional associations and interventions implemented to improve body image and quality of life. *Materials and Methods:* We conducted a literature search from 1 January 2018 to June 2021 that included electronic searches of six major databases (PubMed, ScienceDirect, ProQuest, PsycINFO, PsychArticles and Scopus) and review of references of articles screened. Of 620 records, only 9 articles met the eligibility criteria. *Results:* Numerous studies have been conducted to analyze various psychological variables, but there is still a lack of standardization in the assessment of body image perception (BI) and quality of life, resulting in small-scale testing of interventions with poor results. *Conclusions:* Expected longitudinal studies describing the flow of body image problems and the mediation and balance factors associated with body image will allow researchers to design methods aimed at limiting body image disorders and thus improving quality of life of patients with head and neck cancer.

## 1. Introduction

Most of the major challenges posed by the need for medical care around the world are related to the long-term care of chronic diseases. Diseases that were once fatal are now treatable, but they still have a profound impact on the quality of life of patients and survivors and require ongoing medical care even after recovery. A central problem with chronic illness is emotional distress. The American Psychiatric Association has recognized the diagnosis of cancer as a traumatic stressor because it can lead to impairments in several areas of functioning (ability to work and social relationships) due to negative cognitions and moods [1].

Cancers of the head and neck are a heterogeneous group of cancers, representing the seventh most common cancer in both sexes worldwide in 2018, accounting for 3% of all cancers [2,3,4,5]. This type of cancer occurs in cosmetically and functionally critical areas and results in life-altering disfigurement, difficulty swallowing and speech problems. Traditionally, tobacco and alcohol use have been the main risk factors for head and neck (HNC) in developing countries, and the increasing transmission of human papillomavirus (HPV) in developed countries has led to significantly more HNC cases worldwide [2,3,4,5]. Men have a higher risk of developing HNC than women [3]. Patients with HNC are four times more likely to commit suicide than other cancer patients [4]. In addition, suicide rates were highest in patients with laryngeal cancer and hypopharyngeal cancer [4].

The devastating impact of HNC on quality of life (QoL) is determined by the fact that *“the face”* is an important component of personality, self-image and interpersonal relationships [6]. Physically, the face is the most exposed, conspicuous and visible part of the body, uncovered by clothing in most people, and conveys individual identity. Functionally, it inspires intellect, communication and emotion, or the representation of the self. Cognitively, the environment is taken in through the senses of sight, hearing, taste and smell. Emotionally, an attractive face is often associated with well-being. It is also a matter of culture, where beauty, especially in women, is associated with art, social status, personal and collective well-being [6].

As a newly defined but “old as times” concept, body image has a complex meaning in contemporary society and can be defined as a multidimensional dynamic perception of the body itself (somato-perception controlled by body position in space, interoceptive and exteroceptive inputs), which is distinct from cognitive, culturally influenced representation (somato-representation or semantic knowledge about the body) [7,8,9,10]. Moreover, body image is closely related to identity, attractiveness, self-esteem, social relationships, sexual functioning and a number of social aspects that are constantly updated [11]. It largely plays out at an unconscious level and is usually regulated by the state of the body [12]. The primary changes in body image that occur during HNC surgery are caused by facial disfigurement and dysfunction.

Dissatisfaction with one’s body can also affect sexuality [9]. HNC survivors are threatened in all its dimensions: in sexual identity (the highly visible changes in facial shape alter the patient’s ability to show facial expressions, which are important for normal nonverbal communication) and in sexual relationships (e.g., difficulties in relating to a partner due to shame about one’s body) [7].

Disfigurement due to HNC is stigmatized in society because beauty is a social goal, desire and standard. Disfigurement is significantly associated with deterioration of structures related to personal identity, communication skills, social relationships, impaired sexuality and clinical levels of depression and anxiety [13,14,15]. Despite the high risk of body image disturbances in HNC patients, there are no effective treatment options for these particular patients.

HNC and its treatment is often associated with significant morbidity, deformity, loss of function and high treatment costs. In daily life, the negative changes in these patients (e.g., difficulties with appearance, disturbances in swallowing, dental hygiene, digestion, speech, pain, role function, movement and psychological well-being) reported in Patient Concerns Inventory often go beyond the realm of physical health, as the disease can also cause psychological problems and increase social demands [16]. The literature reports that 75% of surgically treated HNC patients experience psychosocial problems [15].

As mentioned earlier, body image (BI) is an important psychosocial issue in head and neck oncology. Head and neck surgeons should play an important role in screening their patients for depression and anxiety. As part of the multidisciplinary team, professionally trained psychologists can assist with formal assessment and refer patients to specialist psychiatrists as needed.

The concept of psychosocial impact is defined as the effects of environmental and/or biological factors on the social and/or psychological aspects of individuals to improve understanding of the impact of disasters on people and communities [17].

Body image is an important aspect of quality of life that has been studied to a very limited extent in patients with HNC. However, a systemic review published in 2018 [18], which examined body image and perceived quality of life in HNC survivors, concluded that body image dissatisfaction is a public health concern due to its high prevalence, as it is often associated with poorer behaviors such as physical inactivity and poor dietary habits, and may be associated with poorer quality of life. In the era of personalized postoperative care for HNC, this study highlights the need to ask patients, caregivers and families what they think are the most important priorities for future research, and it also highlights the need for public health action to address these issues.

The aims of this review were to: (1) assess the impact of HNC treatments on patients’ body image and quality of life; (2) evaluate the relationship between body image and quality of life; and (3) examine interventions implemented to improve body image and quality of life in this population and also identify directions for future research in this important survivorship area.

## 2. Materials and Methods

Publications addressing body image and quality of life in patients with HNC from all geographic regions were identified through systematic searches of PubMed, ScienceDirect, ProQuest, PsycINFO, PsychArticles and Scopus databases from 1 January 2018, to 1 June 2021. The search terms “head and neck cancer”, “body image” and “quality of life” were used in all six databases (Table 1).

### 2.1. Data Collection Process

**Figure 1:**
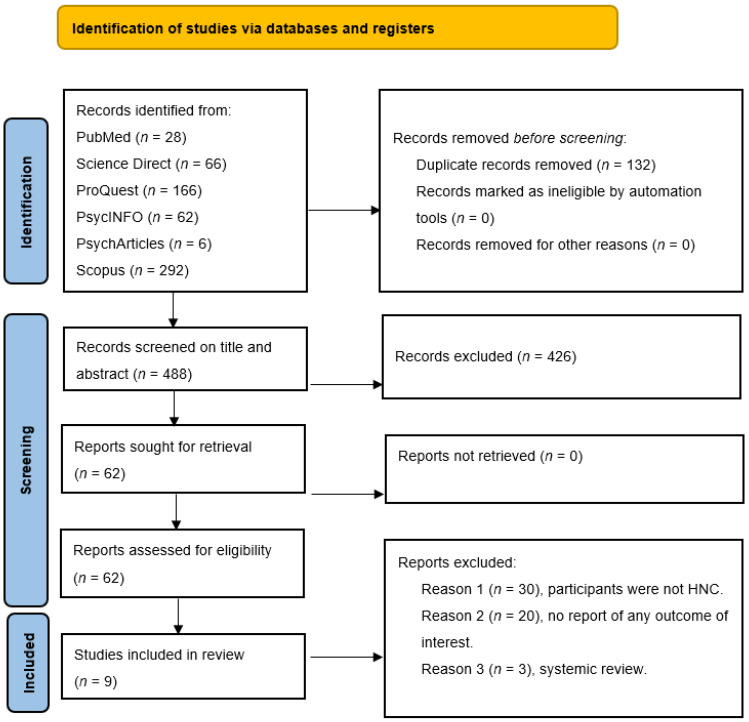
Flow diagram of the selection process of the studies.

After introducing the above keywords and filters regarding publication date and language in all databases, a total of 620 articles were found. Finally, only 9 studies met the eligibility criteria and were included in the systematic review. Figure 1 shows the overview of the selection process using the PRISMA 2020 flowchart, where the data were extracted systematically.

### 2.2. Eligibility Criteria

The inclusion criteria for this review were: (1) original research; (2) published in English as of 2018 with accessible full text; (3) measurement of body image as an outcome variable; and (4) results included reports of age-related outcomes. The definition of “younger” and “older” is not addressed by the reviewers prior to review, as no consensus was reached in the literature on the definition of “young” and “old” in cancer.

Exclusion criteria were: (1) examination of body image with respect to medical imaging; (2) disfigurement due to trauma, burns or congenital health problems, (3) review articles, systemic reviews, unpublished articles, dissertations, commentaries, meeting and conference abstracts, and case reports, book reviews, opinions and editorials.

## 3. Results

According to the search strategy, a total of 620 articles were identified. However, 132 records were duplicated. Of the remaining 488 articles, 426 were excluded after reading titles and abstracts, and of these, 62 were selected based on the eligibility criteria; 53 articles were further excluded (if they did not include HNC survivors, studies that did not evaluate body image and quality of life outcomes, and were systemic reviews), and finally, a total of 9 studies were selected for qualitative synthesis, indicating the scarcity of research in this area. The year 2018 was chosen as the baseline year, as this was when the last paper describing BI and the quality of life of HNC patients was published. The screening process consisted of four steps: (I) title screening; (II) abstract screening; (III) full-text screening; and (IV) critical appraisal. After the initial search, eligible titles and abstracts were analyzed by two authors (V.I.C., D.E.L.), and full-text articles were independently reviewed for eligibility by first and senior authors (V.I.C., V.V.C., B.G.I., R.P.). Three authors searched the reference lists of included publications to identify additional articles (V.V.C., B.G.I., R.P.). Disagreements between authors were resolved by referring back to the original article and discussing with the authors to reach consensus.

### 3.1. Characteristics of the Studies

The general characteristics of the studies included in the review (*n* = 9) can be found in Table 2. The articles were published from 2018 to 2021 in a variety of scientific journals with different aims and scopes: Indian journal of palliative care [19], Supportive care in cancer: official journal of the Multinational Association of Supportive Care in Cancer [20], Sexual Medicine [21], Psycho-oncology [22], Otolaryngology-head and neck surgery: official journal of American Academy of Otolaryngology-Head and Neck Surgery [23], Indian journal of palliative care [24], Psycho-oncology [25], Surgery [26], Medicina oral, patología oral y cirugía bucal [27]. All the records were written in English.

#### 3.1.1. Design of the Studies

Six of the nine studies were descriptive, cross-sectional studies with pre/post design. Only one of the studies was a controlled trial. Thus, it was a quasi-experimental pretest-posttest and follow-up design.

#### 3.1.2. Participants and Regrouping

A total of 1445 HNC patients were enrolled in the studies. The number of participants in the different studies ranged from 10 to 768 HNC patients. Study 1 [19] included 60 HNC patients (46 males/14 females), the average mean age of the patients was 43 years, who were divided into a total of 8 HNC localization groups: carcinoma of buccal mucosa (*n* = 24), carcinoma of tongue (*n* = 14), carcinoma of maxilla (*n* = 3), hard palate (*n* = 3), carcinoma postcricoid/supraglottic (*n* = 5), carcinoma of central arch /mandible (*n* = 6), carcinoma esophagus (*n* = 4) and carcinoma of lip (*n* = 1) in the Department of Pain and Palliative Medicine, Gujarat Cancer and Research Institute, India. Study 2 [20] included 87 patients, with a mean age of 66 years, divided into a total of 5 groups: oral cavity (*n* = 17), oropharynx (*n* = 20), hypopharynx (*n* = 5), larynx (*n* = 29) and others (*n* = 16), from the Department of Otolaryngology—Head and Neck Surgery at Amsterdam UMC, location VUmc. Study 3 [21] consisted of 134 HNC survivors (males = 44/females = 23), with a mean age of 66 years, who were divided into a total of 3 HNC localizations: lip/oral/cavity/oropharynx (*n* = 29), hypopharynx/larynx (*n* = 21), other head and neck cancers (*n* = 17), randomized to investigate differences in the course of sexual interest and sexual pleasure between a care program targeting psychological distress compared to usual care and a control, at the clinic of Amsterdam University Medical Centers (Amsterdam UMC), location VU University medical center. Study 4 [22] is registered at ClinicalTrials.gov (NCT03518671) and was conducted with 10 HNC survivors. Participants were predominantly female (*n* = 7)/male (*n* = 3), had oral cancer (*n* = 4), underwent microvascular reconstruction (*n* = 8) and received adjuvant therapy (*n* = 7) with BID, they were enrolled in a single-arm pilot trial designed to evaluate the feasibility, acceptability and preliminary clinical effect of BRIGHT (Building a Renewed ImaGe after Head and Neck Cancer Treatment). Study 5 [23] enrolled 68 patients (males *n* = 43/females *n* = 25), with a tumor location and histology divided into: oral squamous cell carcinoma (*n* = 37), oropharynx SCC/SCC of unknown primary (*n* = 8), larynx/hypopharynx SCC (*n* = 9) and facial cutaneous malignancy (*n* = 21), with pretreatment and 1, 3-, 6-, 9- and 12-months posttreatment and underwent microvascular reconstruction (*n* = 45); the median age was 63 years. Study 6 [24] included a total of 105 patients (male = 78/ female = 27) who were divided into a total of 6 HNC localizations: oral cavity (*n* = 13), nasopharynx (*n* = 6), oropharynx, (*n* = 60), larynx (*n* = 9), other (*n* = 7), unknown (*n* = 10) from the Henry Joyce Cancer Clinic in the Vanderbilt-Ingram Cancer Center. Study 7 [25] included a total of 168 patients aged over 30 years diagnosed with breast, cervical, head and neck, gastrointestinal tract, lung or colorectal cancer at stage III or IV and who had undergone radiotherapy, chemotherapy or surgery or a combination of both, from a total of 12 hospitals in the southern part of Karnataka (Manipal, Mangalore and Bengaluru). Study 8 [26] was performed with 1710 thyroid cancer survivors (male = 199/female = 1511), who were surveyed online, with the mean age at survey 51 years, mean age at diagnosis 44 years and mean time since surgery 6,8 years. Study 8 [26] was conducted with 1710 thyroid cancer survivors (male = 199/female = 1511) who were interviewed online. The mean age at interview was 51 years, the mean age at diagnosis was 44 years and the mean time since surgery was 68 years. Study 9 [27] included a total of 103 individuals (male = 81/female = 22) with HNC and ages ranging from 20.0 to 81.6 years, with tumor location and histology subdivided as follows: oral cavity (*n*=35), larynx (*n*=26), skin (*n*=16), pharynx (*n* = 12), others/unknown (*n* = 14).

### 3.2. Changes in Body Image after Head and Neck Cancer

Numerous instruments are already in use to assess various body image and quality of life variables. Among the most common are: Body Image Scale (BIS); Body Image Quality of Life (BIQL); Inventory Vanderbilt Head and Neck Symptom survey; European Organization for Research and Treatment of Cancer, Quality of Life Questionnaire, Head and Neck Cancer -specific module, etc.; others are currently being tested, but there is still no validated, comprehensive and efficient tool to correctly assess the impact of HNC on body image and quality of life and predict its future evolution.

As Chang and colleagues [25] found in their study, overall negative body image was associated with higher levels of depression, greater anxiety about social interactions, poorer social-emotional functioning, receipt of surgery, female gender and greater avoidance of social interactions. Body image is an important issue when it comes to patient acceptance of therapies and procedures. For example, in the study by Umrania et al. [19], 88.33% of those who refused a naso-gastric tube (NG) for feeding justified that “it will disrupt my body image”, and also 80% said that they would be “unable to go outside/mix with people” (80%).

Melissant et al. [20] found in their intervention study that expressive writing, an exercise that performed well in people with breast cancer, had no effect on body image disturbance in people with HNC.

An interesting ongoing study, called BRIGHT (Building a Renewed ImaGe after Head and Neck Cancer Treatment) is testing a novel telemedicine-based cognitive-behavioral intervention to manage body image disturbance (BID) in head and neck cancer survivors [22]. In a pilot trial of this study, the 10 patients included presented improved image-related coping behavior [22]. In a pilot trial of this study, the 10 participating patients demonstrated improved image-related coping behaviors [22].

#### Severity of Body Image Disturbance over Time

There were variable findings with regards to the association between the severity of body image disturbance (BID) and time since treatment. Unfortunately, none of the analyzed studies compared pre-to posttreatment BID severity. In general, surgically treated HNC survivors declared to have a disturbed body image after the diagnosis of the disease and early in the survivorship period, but the degree of disturbance varies with the type of surgical procedure. Women were more emotionally affected than men [24,25].

Disfigurement of the head and neck due to surgical cancer treatment was significantly related to the functional dimension of patients’ quality of life, especially in cases of major neck and lower facial sequelae. Patients with HNC who undergo surgery usually undergo further reconstruction 6 to 12 months after the original reconstruction to improve shape, contour, appearance and function.

On the other hand, the BID may remain stable and/or improve over time, especially with continuous adjustment and surgical/flap revisions. There were significant limitations in the methods in many studies examining BID and quality of life in different areas as most of the studies were randomized (with patients in different areas for continuous treatment). These methodological limitations preclude knowledge of the longitudinal course of BID and QoL in patients with HNC. The knowledge gap about the longitudinal course of BID and QoL in patients with HNC, particularly in long-term survivors, precludes delivery of optimally timed, patient-centered preventative and therapeutic interventions for BID. 

Graboyes et al. [23] have shown in one of their studies that patients treated surgically for HNC recover to pretreatment levels of body image dissatisfaction by 9 months posttreatment.

### 3.3. Quality of Life

*“Sexuality and sexual”* needs are often overlooked, even though they are an essential component of quality of life. Only a limited number of studies have examined sexuality in patients with HNC. These studies suggest that HNC and its treatment negatively affect these aspects of sexuality, especially immediately after oncologic treatment and particularly in patients with high levels of distress, impaired social functioning, severe disfigurement and advanced tumor stages [24,25,26]. Moreover, sexuality was mentioned by patients with HNC among the top 3 most distressing areas of their lives. 

Using the European Organization for Research and Treatment of Cancer, Quality of Life Questionnaire, Head and Neck Cancer–specific module, Schutte et al. [21], found in their study that 76.1% of participants had an unmet sexual need at baseline, and 24.6% had a psychiatric disorder (anxiety or depression).

In a study of 103 participants, Noguiera and colleagues [27] used the Functional Assessment questionnaire from Cancer Therapy (FACT-H&N) and concluded that lower quality of life was associated with sequels in the neck and/or lower third of the face, higher degree of disfigurement and female gender. The association between facial disfigurement and quality of life was significantly greater in women, concerning the social and familiar, functional and head and neck cancer specific domains. One of the conclusions of this study is that there is no clear evidence of a linear relationship between the level of facial disfigurement and dysfunction and the impact on QoL, which suggests that other emotional and psychosocial factors may play an important role in individual patient’s response.

### 3.4. Body Image and Quality of Life

The quality of life of patients with HNC has also been studied in relationship to body image. It has been suggested that QoL difficulties rank higher in terms of importance than the body image issues [24]. 

The UW-QOLv4 and the EORTC are the commonest reported QoL questionnaires in HNC and both include sections asking about appearance/disfigurement. In general, there are only a few studies where the relationship between QoL and body image is investigated. One of the possible causes is that in many instances the change in body image is considered an aspect of the QoL and it is evaluated with a subscale within the QoL instrument. 

Surprisingly, a study by Burchfield et al. [24] on 105 participants with HNC found no change in body image but altered quality of life (40%) or poor quality of life (15%) and also a high frequency of neuropsychiatric symptoms: restlessness (47.6%), tension (47.5%), decreased motivation (46.7%), distractibility (38.5%), slowed movements (38.5%) and irritability (38.5%), lack of interest in other people (21.9%). The study used Vanderbilt Head and Neck Symptom survey plus the General Symptom Subscale, the Body Image Quality of Life Inventory, Neurotoxicity Rating Scale, the Profile of Mood States and a five-item quality of life measure.

### 3.5. Specific Interventions

An important determinant of the ability of HNC patients to cope with their disease and treatments is the perceived social support (PSS), which can be defined as the extent to which a person believes that his/her needs for support, information and feedback are fulfilled during times of need [28]. From the nine studies used in this review, some specific interventions may be of interest in the near future (Table 3).

Psychological counseling proved useful for 47 of 60 patients who initially refused the nasogastric tube feeding but then understood its benefits and eventually accepted it [19].

Telemedicine-based cognitive-behavioral intervention to manage body image disturbance (BID) in head and neck cancer (HNC) survivors from BRIGHT (Building a Renewed ImaGe after Head and Neck Cancer Treatment) trial presented promising results on coping behavior in the pilot study [22].

My Changed Body (MyCB) is an exercise of expressive writing pre- and post- treatment which showed an improvement in self-esteem, but no effect on body image distress, measured on Body Image Scale [20].

These results suggest that positive perception of a supportive social network may help patients with HNC better cope with the psychological impact of treatment on their body image.

### 3.6. Limitations, Strengths and Future Directions

Limitations of this study should be mentioned. We excluded studies published in languages other than English, which bias our results. We also excluded unpublished articles, dissertations, commentaries, conference proceedings or meeting and conference abstracts, which put our results at risk of publication bias.

The studies described and analyzed here are heterogeneous in nature with respect to country, study design and population, but there was no discussion in relation to the individual healthcare systems. Many studies were cross-sectional in design and included a heterogeneous mix of patients before and after treatment. Therefore, the relationship between key psychological outcomes and body image disturbance/QoL remains unknown. Few studies have examined the relationship between quality of life and body image, possibly because body image is considered an aspect of quality of life and is assessed with a subscale within the quality-of-life instrument.

The main gaps in the study related to BID research related to quality of life in HNC identified in this review include the following: (1) nutritional status is a factor that appears to be related to body image and thus quality of life, but it has not been analyzed, (2) the lack of an HNC-specific BID patient-reported outcome measures (PROM) and thus the reliance on PROMs developed by patient populations and validated differently, (3) the variability of PROMs used to assess BID and the lack of clear points to distinguish BID from “normal” body image concerns, (4) the nature of the relationship between body image disturbance and other psychosocial variables such as depression, anxiety and social isolation in patients with HNC over the course of treatment and during recovery, (5) the lack of evidence-based interventions in the treatment of BID in patients with HNC, (6) the body satisfaction scale was not assessed in our studies.

From the pilot studies presented in the specific intervention section, we learned that targeted psychological interventions are effective in reducing BI issues related to HNC patients. However, results are mixed as to the magnitude of these effects. It seems worthwhile to replicate the findings in larger studies of psychological support for HNC patients.

Finally, even in the face of the above-mentioned limitations, considering the severity of the disease and the significant impact of treatment side effects on HNC patients, the variety of symptoms, the need for objective evaluation scales and the paucity of possible interventions, we believe that future research and trails are expected in order to minimize symptoms and make treatment less exhausting and traumatic.

## 4. Conclusions

Living after receiving a diagnosis of cancer is a difficult task, but moreover living with the scars of the treatment for head and neck cancer may be extremely physically and psychologically exhausting. HNC and its treatment subside in disfigurement, neurological disorder, impaired senses, a wide variety of psychological disorders, ranging from distress to suicidal thoughts. From the results of the analyses, it can be concluded that body image is related to the quality of life of HNC patients.

While mortality after cancer may be decreasing, new morbidities and impacts on QoL and body image are increasingly recognized in HNC patients. These new morbidities have the potential to disproportionately impact the entire life span of patients, and may indirectly affect caregivers and future dependents. In addition to new disability, HNC survivors are at risk for late mortality and readmission, and often require increased health care resources. First studies in the field indicate that over a third of HNC survivors may have new disability between one to three months after HNC diagnosis and treatment, with physical effects and behavioral impacts lasting years in some cases. There is an unmet need to identify HNC patients at risk of long-term sequelae early, to better characterize the new post-HNC morbidities and their impact, define optimal approaches for post-discharge follow-up, and design effective interventions to enhance recovery and maximize quality of life in HNC survivors.

Recent evidence shows that combining psychological therapies with cosmetic and beauty treatments is important in preventing the development of illness concerns and highlights an important growth area for the future of HNC care, where complex decisions are discussed with patients and commitments are made.

### Recommendations for Future Research

Regarding future research directions, more interventions are needed that focus on developing positive BI and self-esteem in this population, as these factors are important for disease progression and may predict psychological functioning and QoL in HNC patients. For example: (1) Since psychosocial problems vary by personality, behavioral patterns, culture and regional background of the patient and family, assessment of these problems requires a large multicenter longitudinal study to allow generalization; (2) Advances in therapeutic interventions targeting sexuality may increase psychological well-being, but it is not yet known whether interventions specifically targeting psychological problems also reduce sexual problems in patients with HNC; (3) Few studies have published data on patient and caregiver experiences of tracheostomy, particularly in the community. There is a need to better understand these experiences in order to formulate strategies and provide resources to improve the quality of care and overall quality of life for patients with tracheostomy and their caregivers in the hospital and community. (4) Understanding the structure and dynamics of personality should be a priority to assist these types of patients and potentially improve outcomes. Even though some authors have investigated the relationship between body image and quality of life, the role of personality traits in the deterioration of quality of life associated with HNC treatments has no received scientific attention. Qualitative studies could be evaluated to assess patients’ individual perceptions with the aim of exploring deviations from body image and implementing personalized psychological interventions focused on the disease experienced. (5) Many questions related to specific interventions for HNC remain unanswered, e.g., (a) How might interventions to improve body image using technology impact body image in HNC patients? (b) Does social media have an impact on HNC patients’ body image and how they perceive our own appearance? (c) How does cross-cultural perspective impact HNC survivors’ body image?

## Figures and Tables

**Table 1:** Data sources and search strategies.

Database	Search Strategies
PubMed	**Search terms:** “head and neck cancer” AND “body image” AND “quality of life”
	**Filters:** English, publication date: from January 2018 to June 2021, species: human, article type: clinical trial, randomized controlled trial
	**Results:** 28 records
	**Relevant:** 12
Science Direct	**Search terms:** “head and neck cancer” AND “body image” AND “quality of life”
	**Filters:** English, publication date: from January 2018 to 2021, research articles, subject areas: medicine and dentistry, access type: open access, HNC relevant publications
	**Results:** 66 records
	**Relevant:** 1
ProQuest	**Filters:** English, publication date: January 2018 to 2021, sort by: newest first, source type: include—Scholarly Journals, Working Papers, Trade Journals; exclude: Books, Reports, Dissertations and Theses, Wire Feeds, Magazines, Newspapers
	**Results:** 166 records
	**Relevant:** 1
PsycINFO	**Filters:** English, publication date: from January 2018 to June 2021, sorted by journal article
	**Results:** 62 records
	**Relevant:** 0
PsychArticles	**Search terms:** “head and neck cancer” AND “body image” AND “quality of life”
	**Filters:** English, publication date: from January 2018 to June 2021, sorted by journal article
	**Results:** 6 records
	**Relevant:** 0
Scopus	**Search terms:** “head and neck cancer” AND “body image” AND “quality of life”
	**Filters:** English, publication date: from January 2018 to June 2021, species: humans, publication type: article, subject area: medicine
	**Results:** 292 records
	**Relevant:** 5

**Table 2:** Characteristics of the included studies.

Authors	Year	Design	Number of Participants	Measure of Body Image and QoL	Results	Link
1. Umrania et al. [19]	March 2021	Descriptive, cross sectional, after treatment	60 HNC patients	A questionnaire referring to most common cause for enteral feeding refusal. Variables: QoL Disrupted body image Inability of eating and feeling the tastes.	The reasons for refusal of NG tube were “it will disrupt my body image”(88.33%), “unable to go outside/mix with people” (80%) and “dependency on others for activities”(66.66%).	Survey of Psychosocial Issues of Nasogastric Tube Feeding in Head-and-Neck Cancer Patients (nih.gov)
2. Melissant et al. [20]	March 2021	Descriptive, cross sectional, after treatment	87 HNC patients	Baseline survey on body image-related distress My Changed Body—expressive writing activity Variables: Body image related distress. Self-compassion.	Expressive writing activity does not significantly improve body image-related distress, but likely increases self-compassion	A structured expressive writing activity targeting body image-related distress among head and neck cancer survivors: who do we reach and what are the effects?
3. Schutte et al. [21]	January 2021	Randomized control trial, after treatment	134 HNC survivors	“Sexuality” symptom subscale, part of the European Organization for Research and Treatment of Cancer, Quality of Life Questionnaire, Head and Neck Cancer–specific module. Variables: Psychological distress	76.1% had an unmet sexual need at baseline, and 24.6% had a psychiatric disorder (anxiety or depression). Stepwise care did not reduce problems with sexual interest and pleasure in any of the follow-up measurements	Effect of Stepped Care on Sexual Interest and Enjoyment in Distressed Patients with Head and Neck Cancer: A Randomized Controlled Trial—ScienceDirect
4. Graboyes et al. [22]	December 2020	Clinical trial	10 HNC survivors	BRIGHT (Building a Renewed ImaGe after Head and Neck Cancer Treatment), telemedicine-based cognitive-behavioral intervention to manage BID. Variables: Body image disturbance	BRIGHT was associated with a 34.5% reduction in mean Body Image Scale scores at 1 month, an effect that persisted at 3 months—post BRIGHT.	Evaluation of a novel telemedicine-based intervention to manage body image disturbance in head and neck cancer survivors—PubMed (nih.gov)
5. Graboyes et al. [23]	January 2020	Prospective cohort study	68 patients with treated HNC	Body Image Scale Variables: Body image disturbance	BID worsening after treatment before returning to pre-treatment (baseline) levels 9 months after treatment.	Temporal Trajectory of Body Image Disturbance in Patients with Surgically Treated Head and Neck Cancer (nih.gov)
6. Burchfield et al. [24]	December 2019	Descriptive, cross-sectional, after treatment	105 patients with treated HNC	Body Image Quality of Life Inventory Vanderbilt Head and Neck Symptom survey General Symptom Survey Neurotoxicity Rating Scale Profile of Mood States -Short Form Quality of life Variables: QoL	In addition to lower mean quality of life, it was found that a higher proportion of patients in the high systemic symptoms patient group rated quality of life as poor compared to the low systemic symptoms patient group, emotional and intellectual overall negative	Late systemic symptoms in head and neck cancer survivors
7. Chang et al. [25]	May 2019	Descriptive, cross sectional, after treatment	168 people with oral cavity cancer	HADS (Hospital Anxiety and Depression Scale), LSAS (Liebowitz Social Anxiety Scale) UW-QoL (University of Washington Quality of Life Scale) BIS (Body Image Scale) Variables: Body image Socio-emotional function. Depression. Poor perceived attractiveness Dissatisfaction with body appearance	Negative overall body image was associated with greater degree of depression, greater fear of social interactions, poorer social-emotional function, receipt of surgery, female gender and greater avoidance of social interaction.	Factors influencing body image in posttreatment oral cavity cancer patients—PubMed (nih.gov)
8. Kurumety et al. [26]	June 2018	Descriptive, cross sectional, after treatment	1710 thyroid cancer survivors	Online survey with 5-point Likert scale. QoL evaluated through Patient-Reported Outcomes Measurement Information System Variables: Self-reported appearance	Age >45, >2 years since surgery and higher quality of life were independently associated with better self-reported neck appearance.	Post-thyroidectomy neck appearance and impact on quality of life in thyroid cancer survivors—Surgery (surgjournal.com)
9. Nogueira et al. [27]	February 2018	Descriptive, cross sectional, after treatment	103 people with HNC	Functional Assessment of Cancer Therapy (FACT-H&N) questionnaire Variables: QoL; Orofacial functioning, Facial disfigurement	The ssymptoms-related domain had the major impact, while emotional domain was the least affected (79.1% of the maximum possible score)	Factors associated with the quality of life of subjects with facial disfigurement due to surgical treatment of head and neck cancer

**Table 3:** Specific interventions to improve body image and quality of life in people with head and neck cancer.

Authors	Date of Publication	Type of Study	Number of Patients	Intervention	Results	Link
Melissant et al. [20]	March 2021	Descriptive, Cross sectional, after treatment	87 HNC patients	Baseline survey on body image-related distress My Changed Body—expressive writing activity	Expressive writing activity does not significantly improve body image-related distress, but likely increases self-compassion.	A structured expressive writing activity targeting body image-related distress among head and neck cancer survivors: who do we reach and what are the effects?
Umrania et al. [19]	March 2021	Descriptive, cross sectional, after treatment	60 HNC patients	A questionnaire referring to most common cause for enteral feeding refusal. Variables: QoL Disrupted BI Inability of eating and feeling the tastes.	The reasons for refusing NG tube were “it will disrupt my body image”(88.33%), “unable to go outside/mix with people”(80%) and “dependency on others for activities”(66.66%).	Survey of Psychosocial Issues of Nasogastric Tube Feeding in Head-and-Neck Cancer Patients (nih.gov)
Graboyes et al. [22]	December 2020	Ongoing Clinical trial (started on 13th of June 2020, estimated completion date July 2022)	10 HNC survivors	BRIGHT (Building a Renewed ImaGe after Head and Neck Cancer Treatment), telemedicine-based cognitive-behavioral intervention to manage BID.	BRIGHT was associated with a 34.5% reduction in mean Body Image Scale scores at 1-month post-, an effect that was durable at 3-months post-BRIGHT	Evaluation of a novel telemedicine-based intervention to manage body image disturbance in head and neck cancer survivors—PubMed (nih.gov) Building a Renewed ImaGe After Head and Neck Cancer Treatment (BRIGHT) 2.0—Full Text View—ClinicalTrials.gov

## Data Availability

The data presented in this study are available in the main text.
